# Enhancing *Agrobacterium*-mediated plant transformation efficiency through improved ternary vector systems and auxotrophic strains

**DOI:** 10.3389/fpls.2024.1429353

**Published:** 2024-07-23

**Authors:** Ephraim Aliu, Qing Ji, Anna Wlazlo, Sehiza Grosic, Mercy K. Azanu, Kan Wang, Keunsub Lee

**Affiliations:** ^1^ Department of Agronomy, Iowa State University, Ames, IA, United States; ^2^ Crop Bioengineering Center, Iowa State University, Ames, IA, United States; ^3^ Interdepartmental Plant Biology Major, Iowa State University, Ames, IA, United States; ^4^ Department of Plant Genetics, Breeding and Biotechnology, Institute of Biology, Warsaw University of Life Sciences, Warsaw, Poland

**Keywords:** allelic exchange mutagenesis, homologous recombination (HR), INTEGRATE system, maize transformation, ternary vector system

## Abstract

*Agrobacterium*-mediated transformation is an essential tool for functional genomics studies and crop improvements. Recently developed ternary vector systems, which consist of a T-DNA vector and a compatible virulence (*vir*) gene helper plasmid (ternary helper), demonstrated that including an additional *vir* gene helper plasmid into disarmed *Agrobacterium* strains significantly improves T-DNA delivery efficiency, enhancing plant transformation. Here, we report the development of a new ternary helper and thymidine auxotrophic *Agrobacterium* strains to boost *Agrobacterium*-mediated plant transformation efficiency. Auxotrophic *Agrobacterium* strains are useful in reducing *Agrobacterium* overgrowth after the co-cultivation period because they can be easily removed from the explants due to their dependence on essential nutrient supplementation. We generated thymidine auxotrophic strains from public *Agrobacterium* strains EHA101, EHA105, EHA105D, and LBA4404. These strains exhibited thymidine-dependent growth in the bacterial medium, and transient *GUS* expression assay using Arabidopsis seedlings showed that they retain similar T-DNA transfer capability as their original strains. Auxotrophic strains EHA105Thy- and LBA4404T1 were tested for maize B104 immature embryo transformation using our rapid transformation method, and both strains demonstrated comparable transformation frequencies to the control strain LBA4404Thy-. In addition, our new ternary helper pKL2299A, which carries the *virA* gene from pTiBo542 in addition to other *vir* gene operons (*virG*, *virB*, *virC*, *virD*, *virE*, and *virJ*), demonstrated consistently improved maize B104 immature embryo transformation frequencies compared to the original version of pKL2299 (33.3% vs 25.6%, respectively). Therefore, our improved *Agrobacterium* system, including auxotrophic disarmed *Agrobacterium* strains and a new ternary helper plasmid, can be useful for enhancing plant transformation and genome editing applications.

## Introduction

*Agrobacterium tumefaciens* is a widely used plant biotechnology tool for genetic transformation and crop improvement (Gelvin, 2003). The unique ability of *Agrobacterium* to transfer its DNA fragment (transfer DNA or T-DNA) into host cells has been widely utilized for efficient delivery of genes of interest into plant genomes (Gelvin, 2003, 2017; Tzfira and Citovsky, 2006). Virulence (*vir*) genes encoded on the tumor-inducing (Ti) plasmid and chromosome (*chv* genes) enable *Agrobacterium* strains to sense the signals exuded from wounded plants, such as low pH, simple sugars, and phenolic compounds, to initiate the T-DNA delivery process (Gelvin, 2003). Natural T-DNAs carry the biosynthesis genes for plant hormones to induce crown gall tumor formation and opine biosynthesis genes to produce low molecular weight compounds (opines) that are used as an important energy source by the infecting *Agrobacterium* (Tempé and Petit, 1982; Britton et al., 2008). T-DNAs are flanked by two nonidentical repeat sequences called left and right borders (LB and RB), and they can be provided *in trans* on a binary vector for easy manipulation (An et al., 1988; Lee and Gelvin, 2008).

For plant transformation purposes, *Agrobacterium* strains and vector systems have been engineered for several vital aspects. Initially, the Ti plasmids were ‘disarmed’ by deleting the natural tumor-inducing T-DNAs (Ooms et al., 1982; Koncz and Schell, 1986). For instance, strain GV3101 (pMP90) and LBA4404 were produced by disarming C58 and Ach5, respectively. Then, supervirulent Ti plasmids, such as pTiBo542 (Komari et al., 1986), were transferred to different chromosomal backgrounds including C58, resulting in highly virulent *Agrobacterium* strains such as A281 (Sciaky et al., 1978). At the same time, the development of the T-DNA binary vector system greatly simplified cloning procedures, thereby gaining popularity within the research community (Hoekema et al., 1983; An et al., 1988). Today, commonly used ‘artificial’ *Agrobacterium* strains such as EHA101 and EHA105 are disarmed versions of the hypervirulent strain A281 (Hood et al., 1986, 1993). For monocot transformation, the development of super-binary vectors that carry extra copies of *vir* genes on the T-DNA vector significantly improved transformation efficiency and expanded amenable genotypes (Ishida et al., 1996; Komari et al., 2006). More recently, a ternary vector system was developed to avoid the complex cloning procedure and inefficient co-integration step in the super-binary vectors (Anand et al., 2018; Zhang et al., 2019; Kang et al., 2022), while incorporating additional *vir* genes to further boost transformation frequency (Anand et al., 2018). Additionally, the *recA* recombinase gene has been inactivated to reduce unwanted plasmid DNA rearrangements within the *Agrobacterium* cells (Lazo et al., 1991; Rodrigues et al., 2021; Aliu et al., 2022). Lastly, auxotrophic *Agrobacterium* strains were generated and successfully used for plant transformation without causing the overgrowth issues (Ranch et al., 2012; Tamzil et al., 2021; Prías-Blanco et al., 2022).

The thymidine auxotrophic LBA4404Thy- strain was generated by deleting the *thymidylate synthase* gene *thyA*, rendering it unable to survive without thymidine supplementation in the medium (Ranch et al., 2012). Thymidine auxotrophic *Agrobacterium* cells can be easily removed from the explants after the co-cultivation period using reduced amount of antibiotics, making it more cost-effective and reducing antibiotic toxicity to delicate plant tissues (Pollock et al., 1983; Nauerby et al., 1997). Moreover, *Agrobacterium* strains harboring genome engineering reagents, such as clustered regularly interspaced palindromic repeats (CRISPR) systems and herbicide resistance genes, are much less likely to survive outside of the laboratory environments, easing some of the biosafety concerns. However, auxotrophic versions of the most commonly used public *Agrobacterium* strains, such as EHA101 and EHA105 are unavailable in the research community.

Here, we report the generation of thymidine auxotrophic *Agrobacterium* strains of EHA101, EHA105, and an EHA105 derivative EHA105D (Lee et al., 2013) by allelic exchange mutagenesis of the *thyA* gene. In addition, we used recently developed CRISPR RNA-guided targeted DNA insertion system called “INTEGRATE” (Klompe et al., 2019; Vo et al., 2021; Aliu et al., 2022) to generate thymidine auxotrophic strain LBA4404T1. These auxotrophic strains were unable to grow in the bacterial medium without thymidine supplementation. More importantly, they retained similar capabilities for the T-DNA transfer and can be used for plant transformation. When combined with our ternary *vir* gene helper plasmids, EHA105Thy- and LBA4404T1 demonstrated efficient maize B104 immature embryo transformation that is comparable to LBA4404Thy- strain, indicating that our new thymidine auxotrophic *Agrobacterium* strains and compatible ternary vector systems can be useful for plant transformation and genome editing applications.

## Materials and methods

### Bacterial strains and growth conditions

*Agrobacterium tumefaciens* strains and plasmids used in this study are listed in **Table 1**. Thymidine auxotrophic strain LBA4404Thy- (Ranch et al., 2012) was obtained from Corteva Agriscience Inc. Three *Agrobacterium* strains EHA101 (Hood et al., 1986), EHA105 (Hood et al., 1993), and EHA105D (Lee et al., 2013) were used to generate thymidine auxotrophs via allelic exchange mutagenesis of *thyA*. These three strains have the *A. tumefaciens* C58 chromosomal background. EHA105D was derived from EHA105 by deleting the *atsD* gene (*Atu5157*), which might play a role in *Agrobacterium* attachment to plant cells (Matthysse et al., 2000). EHA105D showed slightly higher maize transformation frequency than EHA105 using the binary vector pTF101.1 (Lee et al., 2013). Thymidine auxotrophic strain LBA4404T1 was generated from LBA4404 (Ooms et al., 1982) by insertional mutagenesis using the INTEGRATE system (Aliu et al., 2022).

**Table 1:** Strains and plasmids used in this study.

Item	Description	Reference
Strains		
EHA101	derivative of A281 (A136/pTiBo542), kanamycin resistant	Hood et al., 1986
EHA101Thy-	*thyA* knockout mutant derived from EHA101	This study
EHA105	derivative of A281 (A136/pTiBo542)	Hood et al., 1993
EHA105Thy-	*thyA* knockout mutant derived from EHA105	This study
EHA105D	*atsD* knockout mutant derived from EHA105	Lee et al., 2013
EHA105DThy-	*thyA* knockout mutant derived from EHA105D	This study
LBA4404T1	*thyA* knockout mutant generated by insertional mutagenesis	This study
LBA4404Thy-	*thyA* knockout mutant used as a positive control for AGROBEST assay	Ranch et al., 2012
Plasmids		
pEA186	INTEGRATE vector with *mCherry* cargo	Aliu et al., 2022
pCBL101-RUBY	T-DNA binary vector for betalain biosynthesis marker *RUBY*	Lee et al., 2023
pKL2128	*thyA* knockout vector with *sacB* and spectinomycin resistance gene	This study
pKL2299	Ternary helper plasmid	Kang et al., 2022
pKL2299A	Ternary helper plasmid with *virA* from Bo542 Ti plasmid	This study
pKL2359	A CRISPR/Cas9 construct with maize *Glossy2*-targeting sgRNA	Kang et al., 2022
pKL2505	INTEGRATE vector with *mCherry* cargo for *thyA* mutagenesis in LBA4404	This study
pKLsacB	Suicide vector derived from pTFsacB with spectinomycin resistance gene	This study
pMKA1	*thyA* knockout vector with *sacB* and kanamycin resistance gene	This study
pTF102	Binary vector with a *gus* gene	Frame et al., 2002
pTFsacB	Suicide vector derived from pK19mobsacB (Schäfer et al., 1994)	This study

Agarose gel electrophoresis, restriction enzyme digestion, and other molecular techniques were conducted according to standard protocols or the manufacturer’s instructions. Small amounts of plasmid DNA were prepared using the QIAprep spin Miniprep Plasmid Kit (Qiagen, Hilden, Germany). DNA purification from agarose gel was done using the QIAEX II gel extraction kit (Qiagen). Restriction enzymes were purchased from the New England Biolabs (MA, USA) and oligonucleotides were synthesized by Integrated DNA Technologies (IA, USA). Standard Sanger sequencing analyses were performed by the DNA Facility at Iowa State University (IA, USA) and whole plasmid sequencing was done at PlasmidSaurus (OR, USA).

### Vector construction

Allelic exchange knockout constructs were made as previously described (Ranch et al., 2012; Lee et al., 2013) using a *sacB*-based suicide vector (Schäfer et al., 1994). Firstly, pTFsacB was made by replacing the multiple cloning site (MCS) of pK19mobsacB (Schäfer et al., 1994). pK19mobsacB was digested with *Hin*dIII and *Eco*RI and then ligated with the annealed oligonucleotides MCS-F1 and MCS-R1 (**Supplementary Table S1**). Secondly, PCR primers were designed using the Primer3 software (Untergasser et al., 2012) to amplify about 800 bp of upstream (UP) and downstream (DN) flanking sequences of *thyA* (*Atu2047*) from *A. tumefaciens* strain C58. PCR amplification was performed using the Phusion high-fidelity DNA polymerase (ThermoFisher Scientific, MA, USA) and *A. tumefaciens* C58 genomic DNA according to the manufacturer’s instruction. A 20 µl PCR reaction mix included 1× Phusion HF buffer, 125 µM dNTPs, 0.5 µM primers, and 0.4 units of Phusion high-fidelity DNA polymerase. Thermocycling conditions were as follows: initial denaturation for 30 s at 98°C, followed by 30 cycles of 10 s at 98°C, 15 s at 63°C, 30 s at 72°C, and a final extension for 5 min at 72°C. thyA-UP and thyA-DN PCR products were then individually cloned into the pJET1.2 cloning vector (ThermoFisher Scientific, MA, USA). Sanger sequencing analyses verified the flanking sequences before they were digested with *Xho*I/*Bam*HI and *Bam*HI/*Sph*I, respectively.

pTFsacB was digested with *Xho*I/*Sph*I and ligated with thyA-UP and thyA-DN fragments to produce the knockout construct, pMKA1 (**Supplementary Figure S1A**). Because EHA101 has kanamycin resistance (Hood et al., 1986), pMKA1 could not be used. Thus, we replaced the kanamycin resistance gene in pTFsacB with a spectinomycin resistance gene to generate pKLsacB. pTFsacB backbone excluding the kanamycin resistance gene cassette was PCR amplified using pKLsacB-F1 and R1 (**Supplementary Table S1**) and assembled with the spectinomycin resistance gene cassette amplified with Spec-F1 and R1 (**Supplementary Table S1**) using pYPQ141D (Lowder et al., 2015) as a template. NEBuilder Hifi DNA assembly cloning kit (New England Biolabs, MA, USA) was used for the final assembly. pKLsacB was then digested with *Xho*I/*Sph*I and ligated with the thyA-UP/thyA-DN fragment cut from pMKA1, resulting in pKL2128 (**Supplementary Figure S1B**). pMKA1 was used for EHA105 and EHA105D, while pKL2128 was used for EHA101 to generate thymidine auxotrophic strains.

To generate thymidine auxotrophic LBA4404T1 strain, oligonucleotides Ach5_thyA_oligo3 and Ach5_thyA-oligo4 (**Supplementary Table S1**) were phosphorylated, annealed, and ligated with *Bsa*I-digested pEA186 (Aliu et al., 2022) to clone a 32-bp spacer targeting *thyA* gene resulting in pKL2505. This construct carries a red fluorescent protein (RFP) gene (m*Cherry*) cassette as a cargo and successful DNA insertion disrupts the target gene and produces RFP-expressing cells.

A new *vir* helper pKL2299A (Addgene #222015) was constructed as follows. The RK2 backbone was amplified by PCR using primers pRK2-F1 and pRK2-R2 (**Supplementary Table S1**) and pKL2299 (Kang et al., 2022) as a template. The *virA* gene cassette was amplified by PCR from AGL-1 genomic DNA using primers Bo542-virA-F1 and Bo542-virA-R1 (**Supplementary Table S1**). All other *vir* genes were obtained from pVS1-VIR1 (Zhang et al., 2019) by *I*-*Sce*I digestion. pKL2299A was made by assembling the RK2 backbone, *virA* gene cassette, and the 26 kb *vir* gene fragment (*virB*, *virC*, *virD*, *virE*, *virG*, and *virJ*) using NEBuilder Hifi DNA assembly master mix following the manufacturer’s instruction (New England Biolabs, MA, USA).

Two T-DNA vectors, pKL2359 and pCBL101-RUBY, were previously reported (Lee et al., 2023). The CRISPR/Cas9 construct pKL2359 carries a guide RNA targeting maize *glossy2* gene and pCBL101-RUBY encodes a visible marker *RUBY* reporter which converts tyrosine into purple pigment betalain (He et al., 2020).

### Generation of *thyA* knockout mutants

*For EHA101, EHA105 and EHA105D*: pMKA1 and pKL2128 (**Supplementary Figure S1**) were introduced into *Agrobacterium* strains EHA101, EHA105, and EHA105D by electroporation as described previously (Mattanovich et al., 1989) using a Bio-Rad Gene Pulser (Bio-Rad, CA, USA). After electroporation, 500 µL of SOC medium was added and incubated in a 28°C incubator for 2 h with shaking at 200 rpm. *Agrobacterium* cells were collected by centrifugation and resuspended in about 100 µL of SOC medium and spread on fresh Yeast Extract Peptone (YEP, 10 g/L Yeast Extract, 10 g/L Bacto™ Peptone, 5 g/L NaCl, pH 7.0, 15 g/L Bacto™ agar) plate amended with appropriate antibiotics. Plates were sealed with parafilm and incubated at 28°C for two days. Antibiotic-resistant colonies (kanamycin-resistant EHA105 and EHA105D; spectinomycin-resistant EHA101) were picked and resuspended in 500 µL of fresh YEP medium in 1.5 mL microcentrifuge tubes and 100 µL was spread on solid YEP medium amended with 5% sucrose and 50 – 150 mg/L of thymidine. Two days later, well-isolated colonies were picked and inoculated on three plates: YEP without thymidine, YEP with 50 mg/L thymidine, and YEP with 50 mg/L thymidine and 50 mg/L kanamycin or 100 mg/L spectinomycin. Colonies that can grow only on YEP with 50 mg/L thymidine plate were screened by PCR using primers DthyA-seq-F1 and DthyA-seq-R1 (**Supplementary Table S1**), and about 274 bp PCR products were subjected to Sanger sequencing to verify precise *thyA* deletion mutants.

*For LBA4404*: The INTEGRATE vector pKL2505 was introduced into LBA4404 competent cells via electroporation as described above. After two days, spectinomycin resistant colonies were collected using an inoculation loop, resuspended in 5 mL of YEP medium, and grown overnight in a shaking incubator at 28°C with 200 rpm. Serial dilutions were made from the overnight culture and about 100 µL cell suspension with 10^6^ dilution was spread on a YEP solid medium amended with thymidine (50 mg/L) and spectinomycin (50 mg/L). Two days later, individual colonies were picked and screened by PCR using primers Ach5_thyA-SF1 and Ach5_thyA-SR1 (**Supplementary Table S1**), and colonies showing pure insertion mutation were resuspended in liquid YEP medium before spreading on solid YEP medium amended with 5% sucrose and 50 mg/L thymidine for *sacB*-mediated curing of the INTEGRATE vector pKL2505. The resulting colonies were tested for spectinomycin susceptibility and thymidine dependent growth.

### Evaluation of T-DNA delivery ability of thymidine auxotrophic strains

We used the *Agrobacterium*-mediated enhanced seedling transformation (AGROBEST) assay (Wu et al., 2014) to test if the *thyA* knockout mutants retain T-DNA delivery capability. *Arabidopsis thaliana* T-DNA insertion mutant *efr-1* (SALK 044334) was obtained from the Arabidopsis Biological Resource Center (OH, USA). About 300-500 seeds were surface sterilized in a 1.5 mL tube by soaking in 1 mL of 50% bleach (3% sodium hypochlorite final concentration) and 0.1% SDS solution for 15 min followed by rinsing four times with sterile water. One milliliter of ½ MS medium supplemented with 5% sucrose was added to each tube, and seeds were transferred to a 60 mm Petri dish using a wide-bore pipette tip and a pipette. The *efr*-1 seeds were subjected to a cold treatment (4°C) for 48 h for synchronized seed germination and grown for 7 days in a growth chamber at 22°C under a 16 h/8 h light/dark cycle.

Thymidine auxotrophic strains and their corresponding wildtype (WT) strains (positive control) were transformed with the binary vector pTF102 (Frame et al., 2002) by electroporation as described above. The binary vector pTF102 carries a *GUS* reporter gene (ß-glucuronidase) driven by a cauliflower mosaic virus 35S promoter. *Agrobacterium* strains were grown for 20 h in 5 mL of YEP medium supplemented with 50 mg/L thymidine (for auxotrophs) and appropriate antibiotics (50 mg/L kanamycin and 100 mg/L spectinomycin for EHA101; 100 mg/L spectinomycin for EHA105 and EHA105D) in 50 mL tubes at 28°C with 200 rpm. Immediately before infection, *Agrobacterium* cells were pelleted by centrifugation and re-suspended in AB induction medium (Gelvin, 2006) to a density of OD_550_ = 0.04.

For *Agrobacterium* infection, about 10 Arabidopsis seedlings were transferred to each well of a 12-well plate using sterile inoculation loops. Five hundred microliters of ½ MS medium was aliquoted to each well before adding an equal volume (500 µL) of freshly prepared *Agrobacterium* cell suspensions supplemented with 50 mg/L thymidine. Each *Agrobacterium* strain was added to three wells (replicates) in each experiment. The 12-well plates were sealed with 3 M micropore tape and incubated in a growth chamber for two days at 22°C under a 16 h/8 h light/dark cycle. After two-day co-cultivation, *Agrobacterium* cells were removed by pipetting, and one milliliter of fresh ½ MS medium amended with 100 mg/L cefotaxime and 100 mg/L timentin were added into each well and Arabidopsis seedlings were further grown for two days in the growth chamber. EHA105 strain without pTF102 was used as negative control.

Transient transgene expression was visualized by GUS staining as previously described with slight modifications (Cervera, 2005). Briefly, the liquid medium was removed, and 1 mL of GUS staining solution was added to Arabidopsis seedlings in a 12-well plate and incubated at 37°C overnight. Following overnight incubation, the GUS staining solution was removed and 75% ethanol was added to the seedlings and left overnight to remove chlorophyll. Arabidopsis seedlings were put on a white background and their images were taken to compare T-DNA delivery efficiencies between the auxotrophic and their corresponding WT strains.

### Thymidine-dependent growth of auxotrophs

Thymidine-dependent growth was monitored in a liquid YEP medium. Seed cultures of thymidine auxotrophs (EHA101Thy-, EHA105Thy-, and EHA105DThy-) and their WT strains were grown in 5 mL of YEP medium in a 50 mL falcon tube in a shaking incubator for 15 h at 28°C with 200 rpm. A batch culture was prepared by transferring a calculated amount of overnight culture to 50 mL of YEP medium in a 250 mL flask to a cell density of 0.02 OD_550_. Batch cultures were grown in a shaking incubator (28°C, 200 rpm), and 0.5 mL of culture was sampled every 2 h for 24 h to measure optical density using a spectrophotometer.

The number of viable cells in the batch culture was monitored for the first 8 h. One hundred microliters of culture was sampled every 2 h and serially diluted. One hundred microliters of the diluted cultures (×10^5^ and ×10^6^) were then spread on solid YEP agar plates supplemented with appropriate antibiotics and 50 mg/L thymidine. Plates were incubated at 28°C for 48 h and the number of colony-forming units (CFU/mL) at each time point was determined.

Thymidine-dependent growth of auxotrophic strains during cocultivation was further evaluated using the AGROBEST assay as described above. For the WT strains, no thymidine was supplemented to the cocultivation medium, whereas auxotrophic strains were grown with or without 50 mg/L of thymine to assess their capability to acquire thymidine from plant tissues or exudes. Initial *Agrobacterium* cell density was adjusted to OD_600_ = 0.02 and final cell density was measured 48 h after the cocultivation.

### Maize B104 immature embryo transformation

Maize transformation was performed using the rapid B104 immature embryo transformation method as previously described (Kang et al., 2022; Lee et al., 2023). *Agrobacterium* strains were transformed with T-DNA constructs pKL2359 (Lee et al., 2023) or pCBL101-RUBY (Lee et al., 2023) that harbors *neomycin phosphotransferase II* gene (*NptII*) for plant selection with or without a ternary helper plasmid, pKL2299 or pKL2299A.

## Results

### Generation of thymidine auxotrophic strains

**Figure 1:**
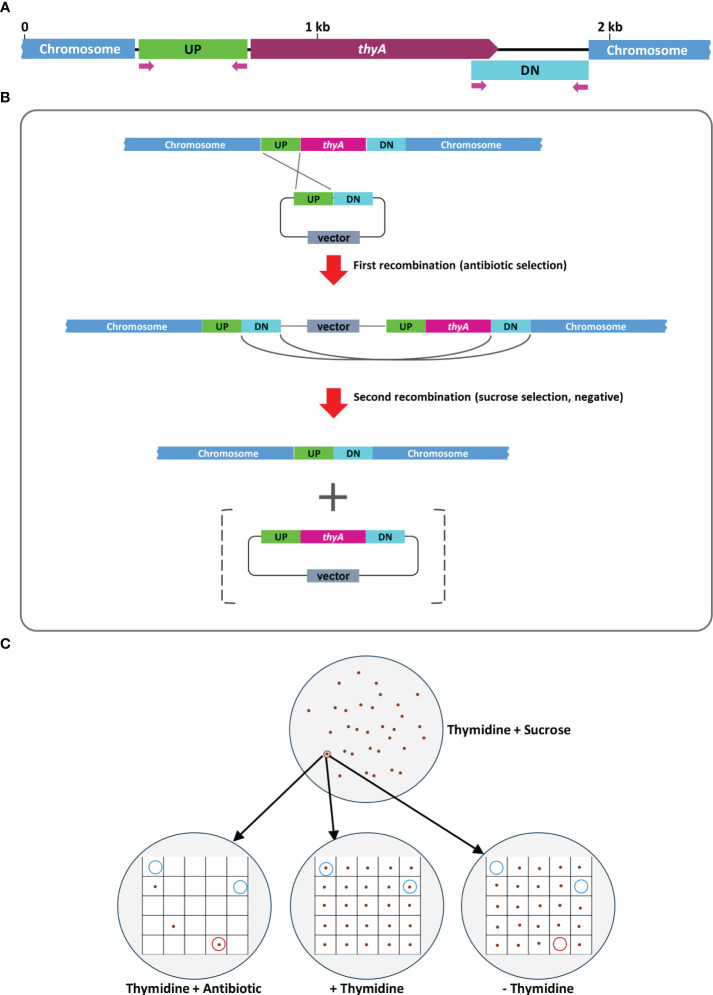
A graphic illustration of homologous recombination mediated *thyA* knockout in *Agrobacterium tumefaciens*. **(A)** Map of the *thyA* region in the circular chromosome of *A. tumefaciens* C58. The upstream (UP) and downstream (DN) flanking sequences of *thyA* and the primers used for PCR are indicated. **(B)** Single homologous recombination between the UP (or DN) sequences leads to integration of the knockout construct into the *Agrobacterium* chromosome. Antibiotic resistance gene encoded on the vector backbone confers resistance during bacterial selection. Antibiotic resistant *Agrobacterium* cells are sensitive to sucrose due to the *sacB* gene encoded on the vector backbone, whose product converts sucrose into levan, a toxic molecule (Gay et al., 1983; Steinmetz et al., 1983). During the negative selection on the antibiotic-free medium containing 5% sucrose and 50-150 mg/L of thymidine, second homologous recombination between the DN (or UP) sequences leads to deletion of the vector backbone and *thyA* gene. The vector containing the *thyA* gene is lost during the negative sucrose selection. **(C)** Sucrose-tolerant *Agrobacterium* cells are tested for antibiotic sensitivity and thymidine dependent growth. Colonies that can grow only on the thymidine supplemented plate (blue circled) are screened by PCR and *thyA* knockout mutation is confirmed by Sanger sequencing analysis.

**Figure 2:**
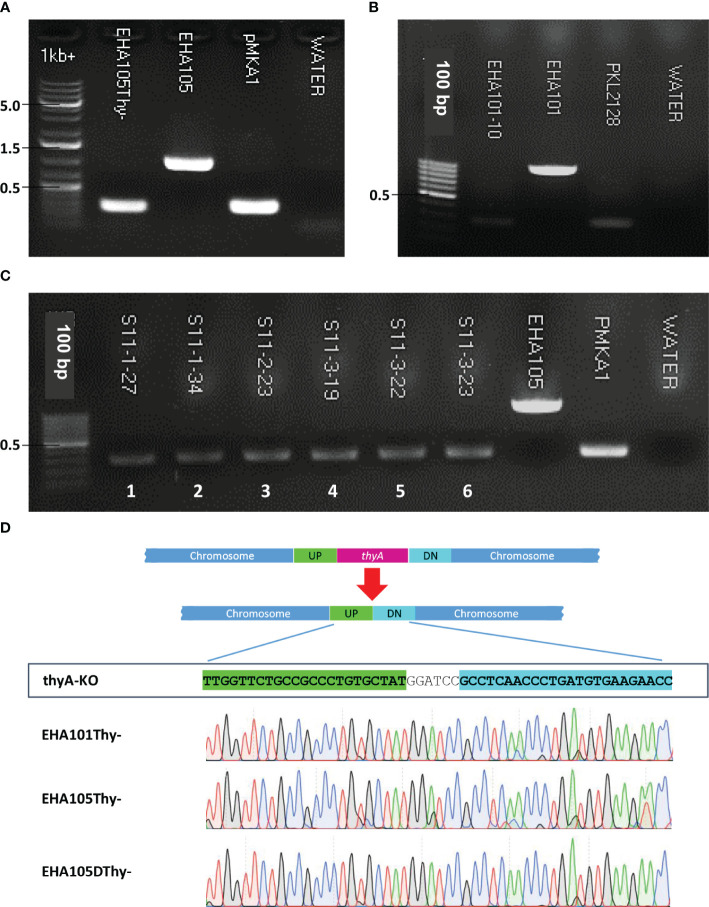
Screening of *thyA* knockout mutants by PCR and Sanger sequencing. **(A)** EHA105Thy-, **(B)** EHA101, **(C)** EHA105DThy-, and **(D)** Sanger sequencing chromatograms showing the *thyA* deletion junctions in EHA101Thy-, EHA105Thy-, and EHA105DThy-. EHA101 or EHA105 were used as WT control (1003 bp), while pMKA1 was used as a knockout control (274 bp). The *thyA* deletion junction of the UP and DN flanking sequences contains the *Bam*HI recognition site (GGATCC) introduced during the knockout vector construction.

The overall procedure of allelic exchange mutagenesis of *thyA* gene is illustrated in **Figure 1**. In the first screening of the EHA105Thy- mutant, we supplemented the YEP medium with 5% sucrose and 50 mg/L thymidine. A total of 120 sucrose-tolerant colonies were screened and only one colony exhibited thymidine-dependent growth without kanamycin resistance. PCR screening amplified an expected 274 bp fragment from the EHA105Thy- colony (1003 bp fragment from the WT EHA105; **Figure 2A**), suggesting it was likely a *thyA* knockout mutant. Similar screening of EHA101Thy- colonies also showed that one *thyA* knockout mutant out of 120 was obtained (**Figure 2B**). The low knockout/WT ratio (1/120) was likely attributed to the lethality of the *thyA* mutation within *Agrobacterium* cells. Therefore, we increased thymidine concentration from 50 to 150 mg/L for EHA105DThy- screening, and obtained six knockout mutants from 120 colonies (6/120), suggesting that increased thymidine concentration in the medium might enhance the survival of the *thyA* knockout mutant cells after the second recombination. Furthermore, PCR screening confirmed that all six colonies carried a *thyA* deletion (**Figure 2C**).

The junction sequences of the *thyA* knockout mutants were subjected to Sanger sequencing to verify the precise sequence deletion by HR. Sequencing results showed that all knockout mutants carried the intended *thyA* deletion mutation (**Figure 2D**). Interestingly, three of the six EHA105DThy- knockout mutants carried an additional 10 bp deletion within the coding sequence of an upstream gene, *Atu2049*, which encodes a transfer-messenger RNA (tmRNA) *SsrA* (**Supplementary Figure S1C**); therefore, we removed these mutant strains from further analyses. It is not clear whether these three mutants were clonal or not, but the presence of an additional mutation in the *thyA* flanking region indicates that even an HR-mediated gene knockout approach can result in unintended mutations. Therefore, a close examination of the junction regions is necessary to avoid unnecessary complication of the downstream analyses.

A thymidine auxotrophic LBA4404T1 strain was generated by inserting *mCherry* cassette into the *thyA* locus using the INTEGRATE vector pKL2505 (**Supplementary Figure S2A**) and following the procedures described previously (Aliu et al., 2022). PCR screening and Sanger sequencing analysis confirmed the targeted insertion of the *mCherry* cassette into the *thyA* locus, 69 bp downstream from the protospacer sequence (**Supplementary Figure S2B**).

### Examination of T-DNA transfer capability

**Figure 3:**
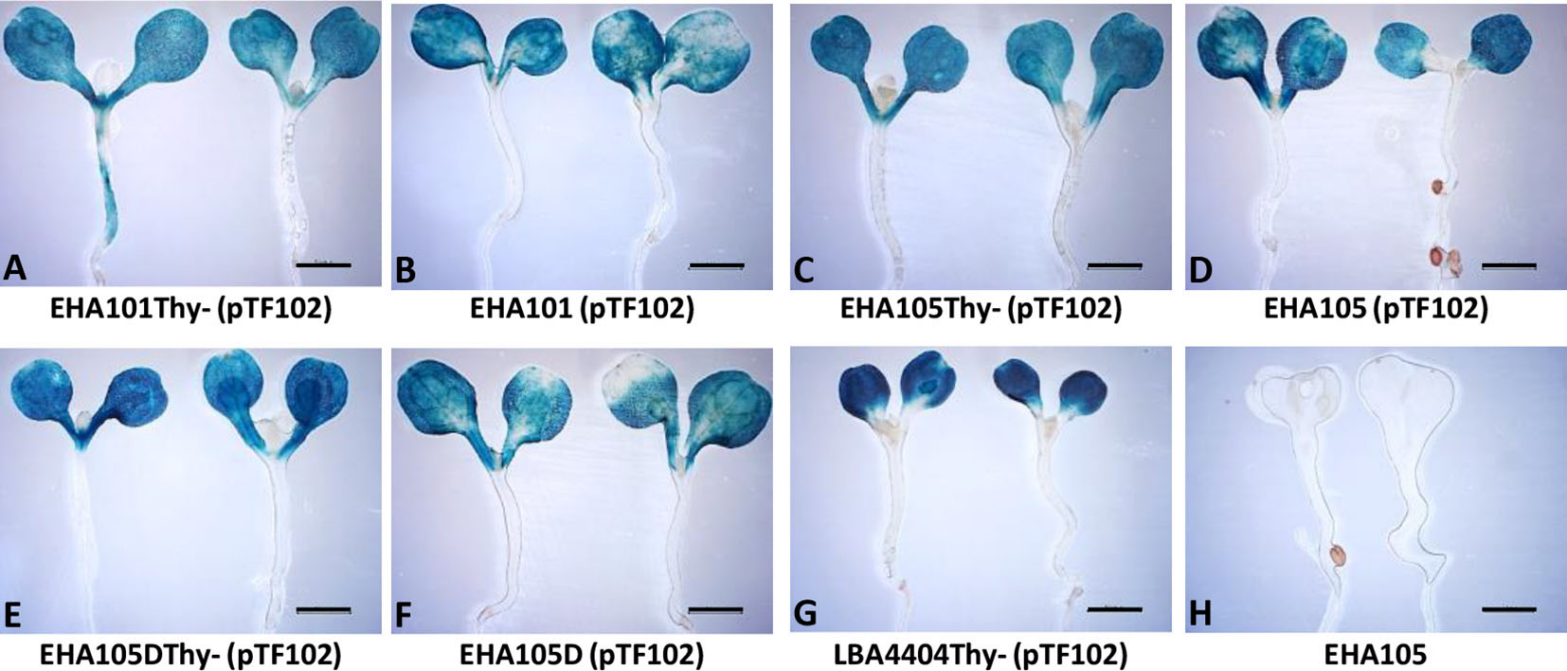
Transient GUS expression assay (AGROBEST) using Arabidopsis *efr-1* seedlings by various *Agrobacterium* strains. Seven-day-old Arabidopsis *efr-1* seedlings infected with different prototrophic and thymidine auxotrophic *Agrobacterium* stains carrying the binary vector pTF102 were compared by GUS staining **(A–G)**. EHA105 strain without pTF102 **(H)** served as a negative control.

We next tested if the thymidine auxotrophic strains can efficiently deliver T-DNAs into plant cells using the AGROBEST assay (Wu et al., 2014). As shown in **Figure 3**, GUS staining results demonstrated that EHA101Thy-, EHA105Thy-, and EHA105DThy- strains retain the T-DNA delivery capability. EHA101Thy- (**Figure 3A**), EHA105Thy- (**Figure 3C**), and EHA105DThy- (**Figure 3E**) strains showed similar level of GUS expression in the Arabidopsis seedlings compared to their corresponding prototrophs (**Figures 3B, D, F**, respectively) and the reference strain LBA4404Thy- (**Figure 3G**). In sum, EHA101Thy-, EHA105Thy-, and EHA105DThy- strains can deliver T-DNA into Arabidopsis cells, and they are ready to be used for transient and stable plant transformation applications.

### Thymidine-dependent growth of auxotrophic *Agrobacterium* strains

**Figure 4:**
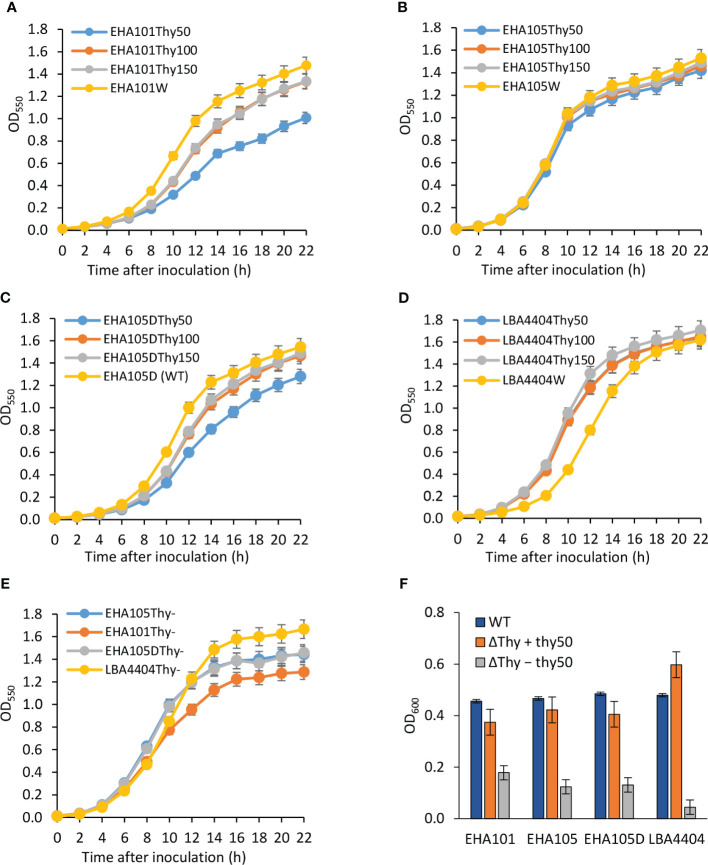
Thymidine-dependent growth of auxotrophic *Agrobacterium* strains. Optical density of *Agrobacterium* cells grown with varying amounts of thymidine was monitored for 22 h at 550 nm using a spectrophotometer. Growth curves were plotted for both the wild-type (WT) strain (yellow curve) and the thymidine auxotrophs at thymidine concentrations of 50 mg/L (Thy50, blue curve), 100 mg/L (Thy100, ash curve), and 150 mg/L (Thy150, orange curve). **(A)** EHA101 and EHA101Thy-, **(B)** EHA105 and EHA105Thy-, **(C)** EHA105D and EHA105DThy-, **(D)** LBA4404 and LBA4404Thy-, **(E)** Average cell density of *Agrobacterium* thymidine auxotrophs with 50 mg/L of thymidine: EHA101Thy- (orange curve), EHA105Thy- (blue curve), EHA105DThy- (ash curve), and LBA4404Thy- (yellow curve). Data represent mean ± standard deviation of three replicates. **(F)** Growth of *Agrobacterium* strains in the cocultivation medium during the AGROBEST assay. *Agrobacterium* strains carrying the binary vector pTF102 were used to infect 7-day-old Arabidopsis *efr-1* seedlings. The optical cell density of the cocultivation medium was measured 48 h after infection at 600 nm using a spectrophotometer. Blue bar (WT) = prototrophs; orange bar (ΔThy + thy50) = thymidine auxotrophs grown in a cocultivation medium supplemented with 50 mg/L of thymidine; and ash bar (ΔThy − thy50) = thymidine auxotrophs grown in a cocultivation medium without thymidine supplementation. Data represent mean ± standard deviation of two replicates.

As all three auxotrophic strains were selected based on their lack of growth on YEP agar medium without thymidine supplement, they did show thymidine-dependent growth in the liquid medium. As mentioned above, increasing thymidine concentration from 50 mg/L to 150 mg/L was helpful in recovering more *thyA* knockout mutants (1/120 vs. 6/120). Additionally, we monitored the growth of each strain in liquid YEP medium supplemented with three different concentrations of thymidine and appropriate antibiotics. As expected, all tested auxotrophic strains showed increased growth rates and maintained higher cell density when supplemented with higher concentrations of thymidine (**Figure 4**; **Supplementary Table S2**). Interestingly, the prototrophic strains grew faster than their corresponding auxotrophic strains, except for LBA4404 strains, whose aggregation in liquid medium made it difficult to accurately measure cell density, even in the presence of 150 mg/L thymidine, suggesting that thymidine uptake might be a limiting factor for the auxotrophic strains. Compared to other strains, EHA101 and EHA101Thy- grew slightly slower, presumably due to the presence of kanamycin in addition to spectinomycin in the medium for other strains.

Overall, the average cell density of the auxotrophic strains across the three different thymidine concentrations (**Figure 4E**) summarized the growth pattern: EHA101Thy- grew slightly slower than EHA105Thy- and EHA105DThy-, which showed nearly identical growth rate over the time course. Lastly, we monitored the number of viable cells from the liquid cultures during the first 8 h of growth (**Supplementary Figure S3**). There was no noticeable difference in the relationship between the optical cell density and the number of viable cells among the auxotrophic and prototrophic strains, further suggesting that these thymidine auxotrophic strains can be properly grown and maintained by supplementing 50 – 150 mg/L thymidine.

Because *Agrobacterium* cells can acquire thymidine from plant tissues or exudes, we monitored how auxotrophic strains and their corresponding prototrophs grow during the cocultivation period using AGROBEST assay to assess their overgrowth potential when co-culturing with Arabidopsis seedlings (**Figure 4F**; Wu et al., 2014). In the experiment, both prototrophic (WT) and auxotrophic versions (ΔThy) of EHA101, EHA105, EHA105D, and LBA4404 strains were tested. The Arabidopsis seedlings cocultivation experiment included the auxotrophic bacterial cultures supplemented with or without thymidine. One-week-old Arabidopsis seedlings were inoculated with *Agrobacterium* cells (initial cell density of OD_600_ = 0.02) and co-cultured for two days. *Agrobacterium* cell density in the cocultivation medium was measured two days after the cocultivation. In a control experiment without Arabidopsis seedlings and thymidine supplement, thymidine auxotrophic strains did not show substantial growth (OD_600_ = 0.04 ~ 0.11). As shown in **Figure 4F**, auxotrophic strains grew well in the presence of 50 mg/L of thymidine (ΔThy + thy50) and reached a cell density (OD_600_ = 0.4 ~ 0.6) that was comparable to their prototrophs (WT, OD_600_ = ~0.5); however, they showed much-reduced growth (OD_600_ = 0.04 ~ 0.18) without thymidine (ΔThy − thy50), demonstrating that thymidine auxotrophic strains indeed have significantly reduced capability to acquire thymidine from the plant tissues or their exudes, and hence a lower risk causing overgrowth during plant transformation.

### Improved *Agrobacterium* system enhances maize B104 immature embryo transformation

We recently established a rapid *Agrobacterium*-mediated maize B104 immature embryo transformation method using a ternary vector system (Kang et al., 2022; Lee et al., 2023). In this study, the thymidine auxotrophic *Agrobacterium* strains were tested for their performance in maize using this method. First, two *vir* helper plasmids were compared using LBA4404Thy- strain. As summarized in **Table 2**, the new *vir* helper pKL2299A (**Supplementary Figure S4**), which carries extra *virA* gene from pTiBo542, consistently outperformed the older *vir* helper pKL2299 (Kang et al., 2022) in each experiment: Exp1, 9% vs. 13.5% (1.5-fold); Exp2, 43.4% vs. 57.8% (1.3-fold); Exp3, 15.8% vs. 22.3% (1.4-fold). Both transient *RUBY* expression and stable transformation frequencies were higher for pKL2299A compared to pKL2299. Although transformation frequencies from each experiment fluctuated due to immature embryo quality variation (Frame et al., 2002; Aesaert et al., 2022), the overall stable transformation frequency of pKL2299A was 33.3%, which is significantly higher than the 25.6% frequency of pKL2299 (*P* < 0.01, two proportion *z* test), suggesting that having the extra copy of the *virA* gene can further enhance T-DNA delivery efficiency, presumably by phosphorylating VirG for activation.

**Table 2:** Summary of B104 transformation using different vir helper plasmids.

Experiment	vir helper	# emb	# RUBY+	% RUBY+	# T0	TF (%)
Exp1	pKL2299	122	109	89.3	11	9.0
	pKL2299A	148	137	92.6	20	13.5
Exp2	pKL2299	196	66	33.7	85	43.4
	pKL2299A	187	107	57.2	108	57.8
Exp3	pKL2299	146	13	8.9	23	15.8
	pKL2299A	148	60	40.5	33	22.3
Total*	**pKL2299**	**464**	**175**	**37.7**	**119**	**25.6 ± 10.5**
	**pKL2299A**	**483**	**244**	**50.5**	**161**	**33.3 ± 13.5**

# emb, number of infected immature embryos; # RUBY+, number of embryos with transient RUBY expression; % RUBY+, percentage of RUBY-positive embryos; # reg, number of regenerated plants; # T0, number of transgenic plants; TF, transformation frequency (# T0 per 100 embryos).

*Values for TF represent mean ± standard error from three replicates.

We then tested if thymidine auxotrophic strain EHA105Thy- can be used for maize transformation with and without the *vir* helper pKL2299. A CRISPR/Cas9 construct pKL2359 (Lee et al., 2023) was used as a T-DNA construct and LBA4404Thy- strain harboring the *vir* helper pKL2299 was used as a control. Interestingly, EHA105Thy- harboring the *vir* helper pKL2299 had a similar transformation frequency compared to the control strain LBA4404Thy- (8.6% vs. 7.8%), whereas EHA105Thy- without the *vir* helper did not generate any transgenic plants (**Table 3**), suggesting that the extra *vir* helper is important for the robust transformation using the rapid B104 method.

**Table 3:** Summary of B104 transformation using EHA105Thy- strain.

Agrobacterium strain	# emb	# calli	# shoot	# R0	# T0	TF (%)
LBA4404Thy-/pKL2299 + pKL2359	128	48	23	11	10	7.8
EHA105Thy-/pKL2299 + pKL2359	128	46	18	13	11	8.6
EHA105Thy-/pKL2359	149	6	0	0	0	0.0

# emb, number of infected immature embryos; # calli, number of embryos with actively growing calli; # shoot, number of calli formed shoots; # R0, number of regenerated plants; # T0, number of transgenic plants; TF, transformation frequency (# T0 per 100 embryos).

Lastly, we tested the new thymidine auxotrophic strain LBA4404T1, generated by insertional mutagenesis using the INTEGRATE system. Our new ternary vector system consisting of pCBL101-RUBY and pKL2299A was used for B104 immature embryo transformation. Both LBA4404T1 and the control strain LBA4404Thy- demonstrated a high efficiency for both transient *RUBY* expression and stable transformation (**Table 4**). Nearly every embryo exhibited betalain pigmentation three days after infection (%RUBY+, **Table 4**) and produced rooted plantlets. While we usually regenerate only one transgenic plantlet per embryo, sometimes we also pick plants that emerge from two distant locations on the same tissue. Taken together, these results indicate that our new thymidine auxotrophic LBA4404T1 strain is useful for plant transformation using the ternary vector system.

**Table 4:** Summary of B104 transformation using LBA4404T1 strain.

Agrobacterium strain	# emb	# RUBY+	% RUBY+	# calli	# shoots	# T0	TF (%)
**LBA4404Thy-**	128	125	97.6	123	149	130	**101.6**
**LBA4404T1**	150	136	90.1	149	186	137	**104.7**

# emb, number of infected immature embryos; # RUBY+, number of embryos with transient RUBY expression; % RUBY+, percentage of RUBY-positive embryos; # reg, number of regenerated plants; # T0, number of transgenic plants; TF, transformation frequency (# T0 per 100 embryos).

## Discussion

*Agrobacterium*-mediated transformation using a ternary vector system is a powerful tool for generating transgenic and gene-edited plants from recalcitrant species or genotypes (Anand et al., 2018). We previously established a rapid maize B104 immature embryo transformation method using QuickCorn media regime (Lowe et al., 2016, 2018; Masters et al., 2020), a thymidine auxotrophic *Agrobacterium* strain LBA4404Thy-, and a ternary *vir* helper pKL2299 (Kang et al., 2022; Lee et al., 2023). pKL2299 contains virulence genes and operons, including *virB*, v*irC*, *virD*, *virE*, *virG*, and *virJ* with their endogenous regulatory sequences originating from the disarmed pTiBo542 (Zhang et al., 2019; Kang et al., 2022). In this study, we produced an improved *vir* helper pKL2299A by adding the *virA* gene isolated from the hypervirulent pTiBo542 (Hood et al., 1986) into pKL2299. The inclusion of the *virA* gene is to ensure that sufficient VirA is expressed to phosphorylate VirG, which subsequently activates other *vir* genes (Gelvin, 2003). Indeed, the new *vir* helper pKL2299A with extra *virA* consistently outperformed pKL2299 with 1.3-1.5-fold higher transformation frequencies. Furthermore, because pKL2299A contains all essential *vir* genes for plant transformation, it can be adopted for both *Agrobacterium* and non-*Agrobacterium* strains for efficient gene delivery applications (Rathore and Mullins, 2018; Cho et al., 2022).

Auxotrophic *Agrobacterium* strains require essential nutrient supplements in the growth media for survival, thus rarely cause overgrowth after co-cultivation, one of the major problems that can significantly reduce plant transformation efficiency (Sutradhar and Mandal, 2023). We successfully generated thymidine auxotrophic strains from *Agrobacterium* strains EHA101, EHA105, EHA105D, and LBA4404 using two different methods. The first approach was allelic exchange mutagenesis of *thyA* gene from the chromosome (Schäfer et al., 1994), and the second method was insertional mutagenesis using CRISPR RNA-guided INTEGRATE system (Klompe et al., 2019; Vo et al., 2021; Aliu et al., 2022). Allelic exchange mutagenesis using a negative selection marker *sacB* relies on homologous recombination (HR) and produces marker-free sequence modifications via a two-step process: knockout construct integration after the first HR and target gene excision after the second HR (**Figure 1B**). Because the HR can occur at either UP or DN sequences, the outcome after the second HR can be either WT or knockout mutant. For essential genes, such as *thyA*, most of the colonies formed after the second HR was WT, thus a large number of colonies were screened to identify a desired mutant (1/120 for EHA101Thy- and EHA105Thy-; 6/120 for EHA105DThy).

On the other hand, the INTEGRATE system provides a simple yet robust approach for targeted mutagenesis in *Agrobacterium* (Aliu et al., 2022). As previously demonstrated by Aliu et al. (2022), thymidine auxotrophic strain was generated from AGL1, which could not be achieved using the allelic exchange mutagenesis due to the *recA* gene mutation, which suppresses HR. However, the INTEGRATE system has some limitations. Compared to HR, which generates scar-free sequence modifications, INTEGRATE-mediated DNA insertions result in the incorporation of extra sequences, including the transposon end sequences (left and right ends) and the 5-bp target site duplication (Klompe et al., 2019; Vo et al., 2021). In addition, repeated insertions into close proximity on a DNA fragment are limited due to the immunity rendered by the existing transposon end sequences (Vo et al., 2021). In this study, we utilized the INTEGRATE system to generate thymidine auxotrophic LBA4404T1 strain by inserting *mCherry* cassette into the *thyA* coding sequence (**Supplementary Figure S2**). Importantly, the thymidine auxotrophic strains generated by either method, i.e., EHA105Thy-, LBA4404Thy-, and LBA4404T1, showed comparable performance in maize B104 immature embryo transformation (**Tables 3**, **4**), indicating that both methods can be applied for *Agrobacterium* strain engineering. Due to the thymidine-dependent growth of the auxotrophic strains (**Figure 4F**), we have not encountered any problems related to *Agrobacterium* overgrowth when using our thymidine auxotrophic strains. Therefore, our generated thymidine auxotrophic strains will be useful for plant genetic transformation without causing overgrowth issues.

In sum, we produced an improved *Agrobacterium* ternary vector system combining auxotrophic strains and a newer version of ternary *vir* helper plasmid for enhancing plant transformation frequencies. This system holds promise for advancing plant transformation and genome editing endeavors.

## Data availability statement

The original contributions presented in the study are included in the article/**Supplementary Material**. Further inquiries can be directed to the corresponding authors.

## Author contributions

EA: Data curation, Formal analysis, Methodology, Validation, Visualization, Writing – original draft, Writing – review & editing. QJ: Data curation, Formal analysis, Methodology, Validation, Writing – review & editing. AW: Data curation, Formal analysis, Methodology, Validation, Writing – review & editing. SG: Methodology, Resources, Writing – review & editing. MKA: Methodology, Writing – review & editing. KW: Conceptualization, Funding acquisition, Project administration, Resources, Supervision, Writing – review & editing, Data curation. KL: Conceptualization, Data curation, Formal analysis, Funding acquisition, Investigation, Methodology, Project administration, Resources, Supervision, Validation, Visualization, Writing – original draft, Writing – review & editing.
