# Supplementary material for: Enhancing Agrobacterium-mediated plant transformation efficiency through improved ternary vector systems and auxotrophic strains
Source: Front Plant Sci. 2024 Jul 23;15:1429353. doi: 10.3389/fpls.2024.1429353 (PMC11300283; doi:10.3389/fpls.2024.1429353)
Supplement: Supplementary file 1 [file DataSheet_1.pdf]

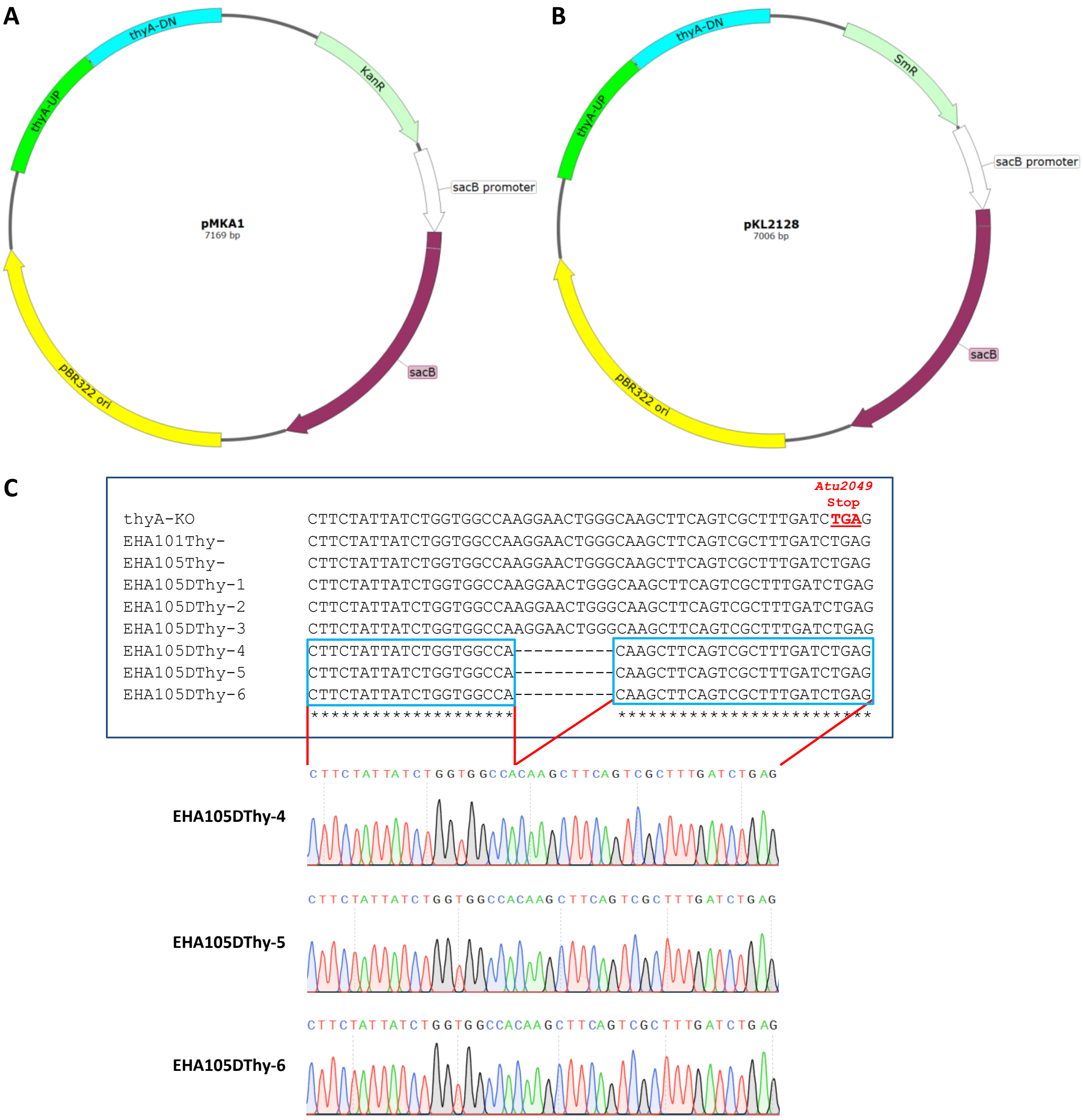

Figure S1. Maps of the *thyA* knockout constructs and sequence alignment of *thyA* junction sequences. (A) pMKA1 has a kanamycin resistance gene and was used for EHA105 and EHA105D. (B) pKL2128 has a spectinomycin resistance gene and was used for EHA101. (C) Multiple sequence alignment of the Sanger sequencing results of the *thyA* UP flanking sequence near the junction. Three mutants (EHA105DThy-4, 5, 6) had a 10 bp deletion within the coding sequence of the *Atu2049*, which encodes a transfer-messenger RNA (tmRNA), *SsrA*.
