# Supplementary material for: Enhancing Agrobacterium-mediated plant transformation efficiency through improved ternary vector systems and auxotrophic strains
Source: Front Plant Sci. 2024 Jul 23;15:1429353. doi: 10.3389/fpls.2024.1429353 (PMC11300283; doi:10.3389/fpls.2024.1429353)
Supplement: Supplementary file 2 [file DataSheet_2.pdf]

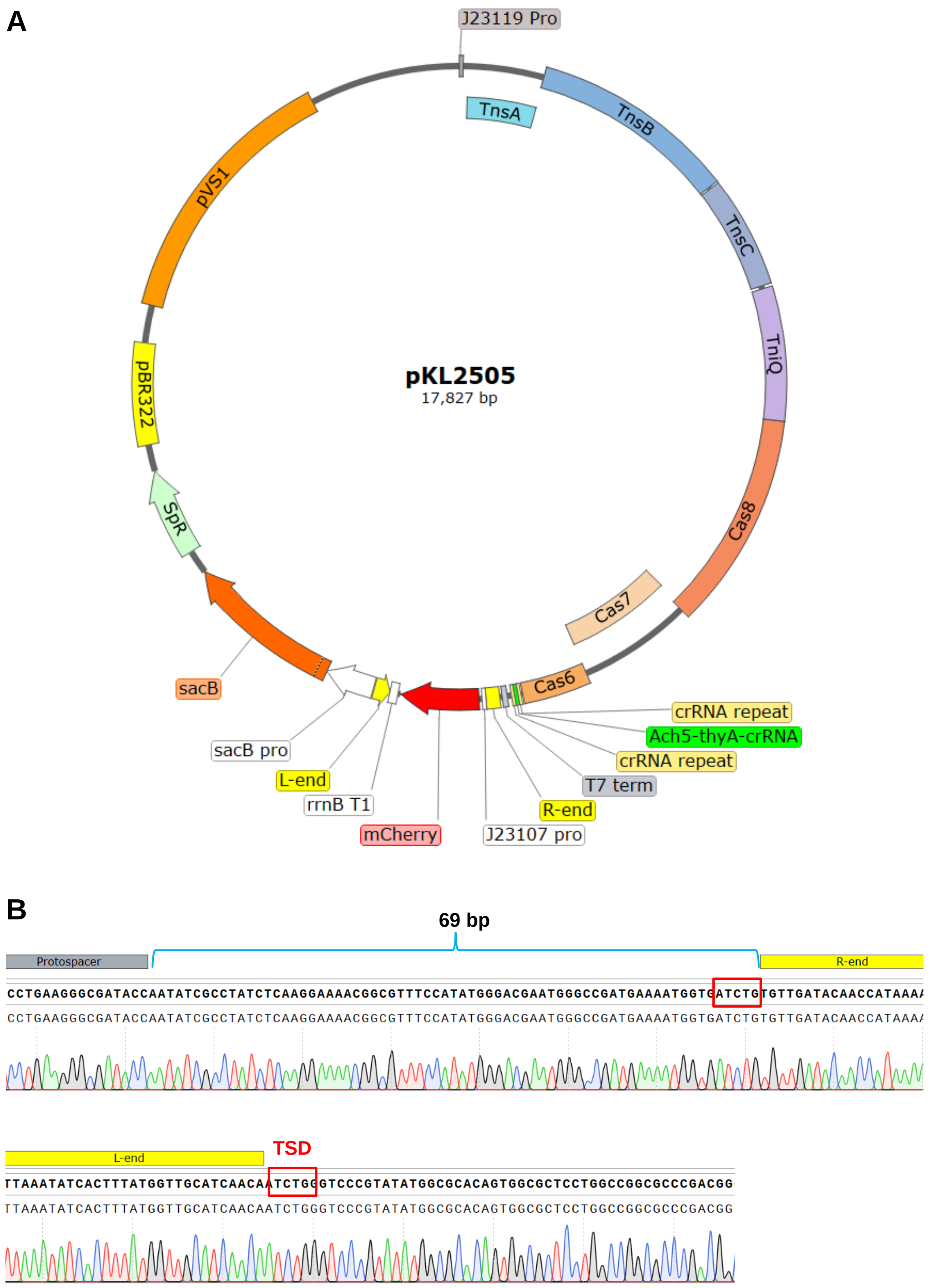

Figure S2. Generation of thymidine auxotrophic LBA4404T1 strain by CRISPR RNA-guided INTEGRATE system. (A) Map of the INTEGRATE vector pKL2505 targeting *thyA* gene. (B) Sanger sequencing analysis confirmed the targeted insertion of the cargo DNA into *thyA* locus: 69 bp downstream from the protospacer sequence. TSD, target site duplication.
