# Supplementary material for: Enhancing Agrobacterium-mediated plant transformation efficiency through improved ternary vector systems and auxotrophic strains
Source: Front Plant Sci. 2024 Jul 23;15:1429353. doi: 10.3389/fpls.2024.1429353 (PMC11300283; doi:10.3389/fpls.2024.1429353)
Supplement: Supplementary file 3 [file DataSheet_3.pdf]

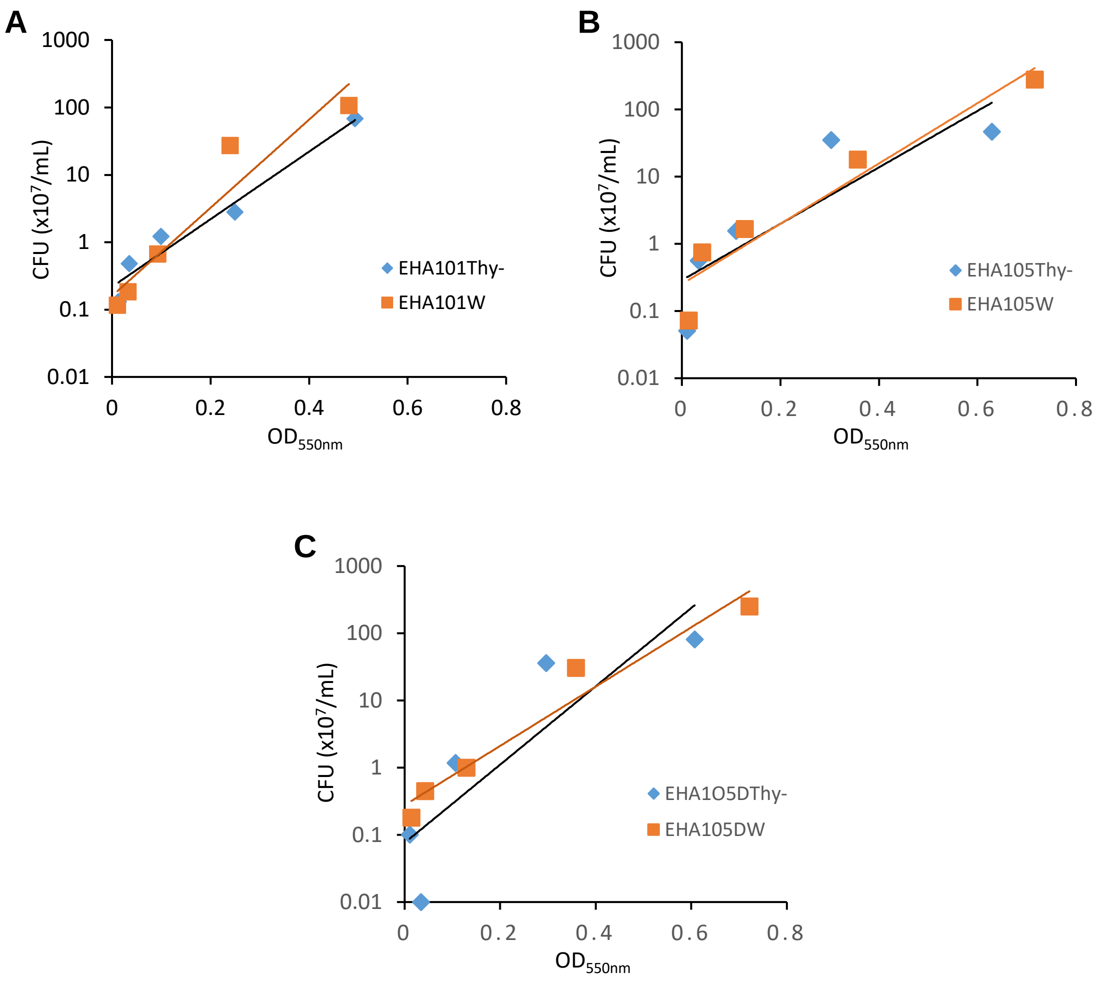

Figure S3. Correlation between the numbers of viable cells (CFU/mL) and optical cell density (OD_{550}). The number of viable cells and optical cell density were measured every two hours for the first 8 hours of growth and the trendlines depict a high level of correlation between them. (A) EHA101 and EHA101Thy-, (B) EHA105 and EHA105Thy-, and (C) EHA105D and EHA105DThy-.
