# Supplementary material for: Enhancing Agrobacterium-mediated plant transformation efficiency through improved ternary vector systems and auxotrophic strains
Source: Front Plant Sci. 2024 Jul 23;15:1429353. doi: 10.3389/fpls.2024.1429353 (PMC11300283; doi:10.3389/fpls.2024.1429353)
Supplement: Supplementary file 4 [file DataSheet_4.pdf]

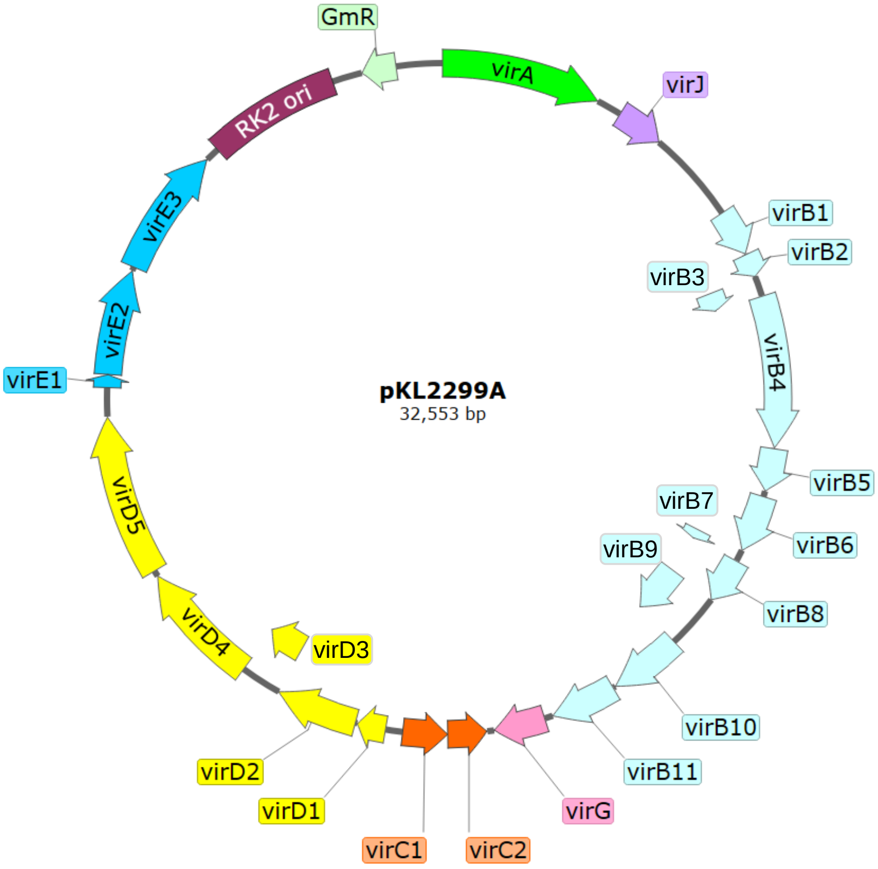

Figure S5. Map of the *vir* helper plasmid pKL2299A (32,553 bp). GmR, gentamicin resistance gene; RK2 ori, origin of replication from the RK2 plasmid; virulence genes and operons (*virA*, *virJ*, *virB1-11*, *virG*, *virC1-2*, *virD1-5*, and *virE1-3*) were originally from the Ti plasmid of *Agrobacterium tumefaciens* Bo542 (pTiBo542).
