# Supplementary material for: Enhancing Agrobacterium-mediated plant transformation efficiency through improved ternary vector systems and auxotrophic strains
Source: Front Plant Sci. 2024 Jul 23;15:1429353. doi: 10.3389/fpls.2024.1429353 (PMC11300283; doi:10.3389/fpls.2024.1429353)
Supplement: Supplementary file 5 [file DataSheet_5.pdf]

Table S1. Oligonucletides used in this study.

Name	Description	Sequence (5'-3')
Ach5_thyA_oligo3	Forward oligo for thyA mutagenesis in LBA4404	ATAACATGAACTCCTGTGGTTCCTGAAGGGCGATACCG
Ach5_thyA_oligo4	Reverse oligo for thyA mutagenesis in LBA4404	TTCACGGTATCGCCCTTCAGGAACCACAGGAGTTCATG
Ach5_thyA-SF1	Forward primer for screening thyA mutants in LBA4404	TTCTCCGGCATGTGATGGAT
Ach5_thyA-SR1	Reverse primer for screening thyA mutants in LBA4404	CACCATCAGCGTCAGAAGTG
Bo542-virA-F1	Forward primer to amplify virA gene from pTiBo542	GGTGGTTGGCATGCACATACACAGCCACAGACAAGTATCGC
Bo542-virA-R1	Reverse primer to amplify virA gene from pTiBo542	CCTGATGGGGTGGCTCTGCATTAGGGATAATTTTCAACCAAACGACCGGAC
DthyA-seq-F1	Forward primer for sequencing	TTCCGATCCAGAAGAGCTTG
DthyA-seq-R1	Reverse primer for sequencing	AGCCGAAAAGGTTCTTCACA
MCS-F1	Forward oligo for multiple cloning site	AGCTTGCATGCATATGCTGCAGTCGACTCTAGAGGATCCCGGGCGGCCGCTCGAG
MCS-R1	Reverse oligo for multiple cloning site	AATTCTCGAGCGGCCGCCCGGGATCCTCTAGAGTCGACTGCAGCATATGCATGCA
pKLsacB-F1	Forward primer for pTFsacB backbone	CCCATCACATATACCTGCCGT
pKLsacB-R1	Reverse primer for pTFsacB backbone	TTGTAAAACGACGGCCAGTG
pRK2-F1	Forward primer to amplify pRK2 backbone	CACCTCATGATGGTCGCGGCTTAGGGATAACCGGTCTTGTCCACTACCTTG
pRK2-R2	Reverse primer to amplify pRK2 backbone	TGTATGTGCATGCCAACCACC
Spec-F1	Forward primer for spectinomycin resistance gene	CACTGGCCGTCGTTTTACAACATCATGCCTCCTCTGGACC
Spec-R1	Reverse primer for spectinomycin resistance gene	CGGCAGGTATATGTGATGGGTGGCTCGAGGGTTATTT
thyA-DN-F1	Forward primer for downstream flanking sequence	GGATCCGCCTCAACCCTGATGTGAAG
thyA-DN-R1	Reverse primer for downstream flanking sequence	GCATGCATAAAAGCCTCTCCCGGTTC
thyA-UP-F1	Forward primer for upstream flanking sequence	CTCGAGATCATGCCGAGCAAAAGTTC
thyA-UP-R1	Reverse primer for upstream flanking sequence	GGATCCATAGCACAGGGCGGCAGA
