# Supplementary material for: Enhancing Agrobacterium-mediated plant transformation efficiency through improved ternary vector systems and auxotrophic strains
Source: Front Plant Sci. 2024 Jul 23;15:1429353. doi: 10.3389/fpls.2024.1429353 (PMC11300283; doi:10.3389/fpls.2024.1429353)
Supplement: Supplementary file 6 [file DataSheet_6.pdf]

Table S2. Doubling time of *Agrobacterium* strains.

Strain	Doubling time (h) during log phase (2-10 h)			
	**Thy50**	**Thy100**	**Thy150**	**WT***
EHA101Thy-	2.40	2.05	2.04	1.89
EHA105Thy-	1.70	1.61	1.64	1.60
EHA105DThy-	2.07	1.91	1.89	1.77
LBA4404Thy-	1.72	1.73	1.69	2.01

*WT: prototroph strain; Thy50, Thy100, Thy150 indicate the concentration of thymidine in the medium, 50, 100, and 150 mg/L, respectively.
